# The plant dehydrin Lti30 stabilizes lipid lamellar structures in varying hydration conditions[S]

**DOI:** 10.1194/jlr.RA120000624

**Published:** 2020-05-13

**Authors:** Jenny Marie Andersson, Quoc Dat Pham, Helena Mateos, Sylvia Eriksson, Pia Harryson, Emma Sparr

**Affiliations:** * Division of Physical Chemistry, Department of Chemistry, Lund University, Lund, Sweden; † Department of Biochemistry and Biophysics, Stockholm University, Stockholm, Sweden

**Keywords:** protein, self-assembly, osmolytes, urea, trimethylamine *N*-oxide, desiccation

## Abstract

A major challenge to plant growth and survival are changes in temperature and diminishing water supply. During acute temperature and water stress, plants often express stress proteins, such as dehydrins, which are intrinsically disordered hydrophilic proteins. In this article, we investigated how the dehydrin Lti30 from *Arabidopsis thaliana* stabilizes membrane systems that are exposed to large changes in hydration. We also compared the effects of Lti30 on membranes with those of the simple osmolytes urea and trimethylamine *N*-oxide. Using X-ray diffraction and solid-state NMR, we studied lipid-protein self-assembly at varying hydration levels. We made the following observations: *1*) the association of Lti30 with anionic membranes relies on electrostatic attraction, and the protein is located in the bilayer interfacial membrane region; *2*) Lti30 can stabilize the lamellar multilayer structure, making it insensitive to variations in water content; *3*) in lipid systems with a composition similar to those present in some seeds and plants, dehydrin can prevent the formation of nonlamellar phases upon drying, which may be crucial for maintaining membrane integrity; and *4*) Lti30 stabilizes bilayer structures both at high and low water contents, whereas the small osmolyte molecules mainly prevent dehydration-induced transitions. These results corroborate the idea that dehydrins are part of a sensitive and multifaceted regulatory mechanism that protects plant cells against stress.

Plants regularly experience osmotic stress that can be caused by drying, freezing, or exposure to aqueous or soil systems with high salt content. Changes in osmotic pressure can lead to changes in biomolecular self-assembled structures, including changes in protein conformation, transformations between different membrane structures, and swelling of multilayer systems. All of these structural changes may have major consequences on biological functions. One example of dehydration-induced changes is found in rye leaves, in which the osmotic stress has been proposed to cause a phase change from planar bilayers to a reversed hexagonal phase in the cell plasma membranes, which may in turn lead to massive leakage and cell injury (1, 2). Another relevant example is dehydration-induced phase segregation and domain formation in cell membranes, which has also been associated with increased membrane permeability (3).

Many plants can counter negative osmotic stress by increasing their tolerance in a process known as acclimation. During acute stress, several different “backup systems” are activated in parallel to help plant survival. One such strategy is to express stress-specific proteins known as late embryogenesis abundant (LEA) proteins (4–7). The LEA proteins are not restricted to plants, and similar proteins can also be found in organisms, including *Escherichia coli*, yeast, and invertebrates (8–10). Another common strategy for protecting biological systems against desiccation relies on the accumulation of small polar molecules with low vapor pressure, commonly called osmolytes. In plants, the osmolytes are most often water-soluble carbohydrates (11–13), while other organisms use different types of osmolyte molecules, including glycerol, urea, and methylamines (e.g., trimethylamine *N*-oxide, or TMAO) (14–16).

Dehydrin proteins constitute a class of intrinsically disordered hydrophilic LEA proteins that are expressed to high levels under conditions of environmental stress such as drought, salt, or low temperatures. The mechanism of how dehydrin proteins protect plants from acute stress is still not clear. Several models have been proposed, including dehydrin stabilizing plasma and organelle membranes (17–21). It has also been suggested that dehydrins act as chaperones or cryoprotectants (22); that dehydrins are hygroscopic, thus preventing complete dehydration (5); and that dehydrin binds metal ions (23). Dehydrins contain a high proportion of hydrophilic and charged amino acids and are characterized by repetitive and highly conserved K-segments (**Fig. 1A**, B). Previous studies have shown that dehydrin Lti30 (K_6_) from *Arabidopsis thaliana* (thale cress) strongly associates with lipid membranes that contain anionic lipids and that the disordered K-segments locally fold up into α-helices on the membrane surface (24–27). The membrane-bound K-segments in these dehydrin proteins are effectively positively charged, and the driving force for the membrane association is likely dominated by electrostatic attraction between positively charged lysine- and histidine-containing segments in dehydrin and negatively charged lipid headgroups (24, 25, 27). The membrane-bound protein may act to protect the membrane structure, and the reported consequences of dehydrin association with lipids include preventing vesicle fusion (20, 28), altering the melting temperature of membrane lipids (21, 24, 28), preventing membrane leakage (20, 22), and bridging between vesicles (24, 25). The majority of these previous studies were performed in excess-solution conditions, whereas the relevant situation of dehydrin-membrane interactions in dehydrated conditions has been far less investigated.

**Fig. 1.**
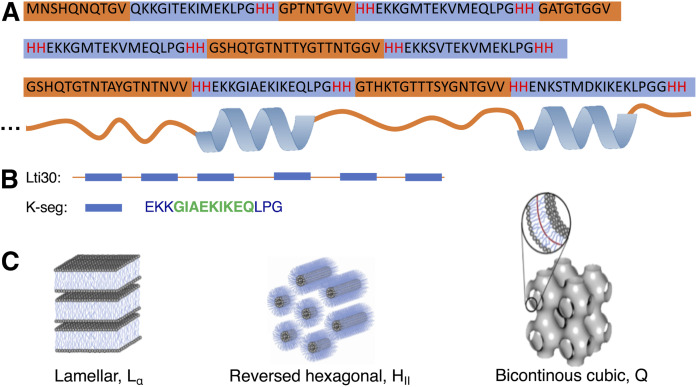
A: Lti30 amino acid sequence with the hydrophobic parts making up the unstructured part of the bound protein marked in orange and the hydrophilic K-segments marked in blue. The histidine amino acids flanking the K-segments are marked in red. B: Schematic representation of the whole Lti30 protein with the K-segments marked as blue squares. The part of the K-segment that folds into an α-helix upon binding with the lipid membrane is marked in green. C: Sketch of the lamellar liquid crystalline, reversed hexagonal, and bicontinuous cubic phase, respectively.

The aim of this study is a molecular understanding of how the dehydrin Lti30 may act to stabilize membrane systems that are exposed to large variations in hydration conditions. It has been previously shown that Lti30 associates with anionic lipid membranes, in which parts of the protein fold into α-helical structures (24–26). Here, we take a membrane perspective, and we study the effects on the molecular properties of the lipids as well as the overall self-assembly membrane structure. We also study how Lti30 influences water uptake and phase transitions in self-assembled lipid systems that are exposed to large variations in water content. In particular, we investigate the possibility that the membrane-bound Lti30 can stabilize such multilayer systems by preventing extensive swelling in water, thereby creating a robust bilayer system that is less sensitive to variations in external hydration compared with the protein-free lipid system. Finally, we compare the effects of dehydrin in varying hydration conditions with that of simple polar osmolyte molecules. The osmolytes used for these studies, urea and TMAO, are typically not found in plants but serve as model compounds to illustrate general molecular mechanisms. Urea and TMAO were chosen as model osmolytes because their effects on lipid membranes in dry conditions have previously been well characterized (29–32).

## MATERIAL AND METHODS

### Lti30 expression and purification

The expression and purification of the recombinant *A. thaliana* dehydrin Lti30 were generally performed as described previously (33) but are presented again for clarification. Lti30 glycerol stocks (150 μl; *E. coli* strain) were spread on Luria agar plates that included 150 μg ampicillin. The plates were then kept at 37°C overnight. The cells were suspended with a small volume of Luria-Bertani medium and added to 2 l Luria-Bertani medium containing 50 μg/ml carbencillin. The cultures were kept at 37°C until an OD_600_ of 0.6–0.7 was reached, whereupon the expression was induced by adding 1 mM isopropyl β*-*d-thiogalactopyranoside. Cultures were then kept at 23°C overnight, after which the cells were harvested by centrifugation at 6,000 rpm for 15 min. The pellet from a 1 l culture was suspended in 25 ml of 20 mM Na_2_HPO_4_ (pH 7.2) and 150 mM NaCl, 1 mM PMSF, and 1 tablet of cOmplete (Roche). To break the cells, these suspensions were sonicated five times for 1 min periods on ice. The larger cell structures were pelleted by centrifugation at 18,000 rpm for 30 min. After this step and to precipitate heat-denatured proteins, the supernatant was placed in an 80°C water bath for 30 min. Heat-denatured proteins were pelleted by centrifugation at 18,000 rpm for 30 min. Lti30 was purified by metal ion affinity chromatography by a standard protocol. The supernatants from heat precipitations were diluted 1:2 with 20 mM Na_2_HPO_4_ (pH 7.2), 1.88 M NaCl, and 1 mM PMSF. The samples were loaded on a 5 ml HiTrap IDA-Sepharose column (GE Healthcare) charged with 7 ml of 3 mg/ml CuSO_4_. The column was equilibrated with 5 vol of 20 mM Na_2_HPO_4_ (pH 7.2) and 1.0 M NaCl. The same buffer (40 vol) was used to wash off the unbound sample from the column, followed by 2 M NH_4_Cl in 20 mM Na_2_HPO_4_ (pH 7.2) and 1.0 M NaCl. Fractions of 5 ml were collected for analysis during the entire run. The column was then equilibrated with 10 vol of 20 mM Na_2_HPO_4_ (pH 7.2) followed by elution of Lti30 with 10 mM EDTA in 20 mM Na_2_HPO_4_ (pH 7.2). To precipitate the Lti30 protein, 80% (NH_4_)_2_SO_4_ was used and left with continuous stirring at 4°C overnight, after which precipitated Lti30 was collected by centrifugation (18,000 rpm for 45 min). Protein pellets were suspended with 2.5 ml of 5 mM MES buffer (pH 6.3) and desalted on PD-10 columns in two steps (GE Healthcare). Lti30 purity was analyzed by the Ready gel SDS-PAGE system (Bio-Rad) (supplemental Fig. S1). Protein quantification was measured with a BCA assay (Sigma-Aldrich). The final protein was freeze-dried and stored at −80°C until use.

### Materials

All lipids were purchased from Avanti Polar Lipids. Urea, TMAO, chloroform, and methanol were obtained from Sigma-Aldrich. Salt solutions used to control the relative humidity (RH) contained potassium chloride, sodium chloride, and KNO_3_, which were purchased from VWR Chemicals, or K_2_SO_4_, which was purchased from Duchefa Biochemie.

### Sample preparation

Stock solutions (40 mg/ml) of each model lipid system were made with chloroform-methanol (2:1). For the preparation of lipid-protein aggregates the chloroform-methanol solution was evaporated by a stream of N_2_ gas into a thin lipid film and left under vacuum overnight. The dry lipid film was dispersed in Milli-Q water and sonicated using either a Qsonica Cup Horn at an amplitude of 70% at an interval of 10:5 for 40 min connected to a chiller set to 40°C (**Table 1**, model D) or a tip sonicator at an amplitude of 50% at an interval of 10:10 for 20 min with the sample immersed in an ice bath to prevent overheating (Table 1, models A–C, E). In all experiments, we used pure Milli-Q water with no added buffer or salt. The size of the vesicles was checked with dynamic light scattering (∼100 nm) using a Zetasizer Nano ZS instrument (Malvern Instruments), and the samples that had been tip-sonicated were centrifuged to remove any residues from the tip. The Lti30 protein was added to the vesicle solution at a concentration of 0.1 mol%. The protein concentration was chosen so that the positive charges of the protein matched the negatively charged lipids corresponding to electroneutral lipid-protein mixtures. The solutions with vesicles and protein were left to equilibrate for 24 h, after which the mixture was centrifuged at 10,000 rpm for 20 min. The supernatant was separated from the pellet, which was washed with Milli-Q water and again centrifuged at 10,000 rpm for 20 min. The supernatant from the wash was separated from the pellet, and both supernatants and the pellet were freeze-dried. The lipid-protein complexes were analyzed with SDS-PAGE to check the integrity of the protein. Dry lipid-protein complexes (∼5 mg) were dispersed in 20 μl of 50 mM glycine buffer (pH 9.0) and mixed with 7 μl SDS-PAGE. The samples were split in half, and one part was boiled. Five microliters each were loaded to a 4% to 20% SDS gel (Bio-Rad).

**TABLE 1.** Lipid model systems used

System	Lipid Composition (Molar Ratio)	Charge	Complex Formation	Structure, Full Hydration	Hydration-Induced Transitions
A	POPC:POPG (95:5)	5 mol% anionic	Yes	L_α_	NA
B	DOPE:DOPC:DOPS (60:35:5)	5 mol% anionic	Yes	L_α_	L_α _→ L_α_/H_II_ → cubic
C	DOPE:DOPC:DOPS (75:20:5)	5 mol% anionic	Yes	L_α_	L_α_/H_II_ → H_II_
D	DOPE:DOPS (95:5)	5 mol% anionic	Yes	H_II_	NA
E	DMPC	Uncharged	No	L_α_	L_α_ → L_β_

DMPC, dimyristoylphosphocholine.

Samples composed of the dry lipid-protein complexes were equilibrated at 27°C in desiccators containing saturated solutions of either sodium chloride (75% RH), potassium chloride (85% RH), KNO_3_ (93% RH) or K_2_SO_4_ (97% RH), or a 5 M sodium chloride solution (∼80% RH) for a minimum of 48 h. For the studies of lipid system A (Table 1), samples were prepared at a controlled water content (wt%) rather than a controlled RH. For these samples, the known amount of Milli-Q water was added to the dry lipid-protein complex, mixed, sealed, and then left to equilibrate at RT for a minimum of 48 h. The NMR and small-angle X-ray scattering (SAXS) measurements were repeated for two independently prepared samples for each composition to confirm reproducibility.

### Solid-state NMR

All NMR experiments were done on natural-abundance ^13^C samples and performed on a Bruker-Avance AVII-500 spectrometer equipped with a Bruker E-free MAS 4 mm probe at ^1^H and ^13^C resonance frequencies of 500 and 125 MHz, respectively. All samples were held in 4 mm inserts and put to a rotor and done under magic angle spinning at a frequency of 5,000 Hz. The inserts were weighed before and after adding the samples to control the amount of sample used in each experiment. The cross-polarization (CP) (34–36), direct polarization (DP), and refocused insensitive nuclei enhanced by polarization transfer (rINEPT) (35, 36) experiments were all used with the same recycle delay, receiver gain, dwell time, number of acquisition points, and decoupling power. The full setup was as follows: acquisition time of 96 ms with a recycle delay of 4 s and 256 scans. Radiofrequency pulses were set to give the nutation frequencies: 80.65 kHz (^13^C 90° and 180° pulses), 80.65 kHz (^1^H rINEPT pulses), 50 kHz (^1^H decoupling pulses), 80–100 kHz (^1^H CP ramp pulse during contact time), and 90 kHz (^13^C CP pulse during contact time). The rINEPT experiments were made with τ_1_ equal to 1.8 ms and τ_2_ equal to 1.2 ms. The CP contact time was 1,000 μs. The experiments were recorded with a spectral width of either 200 or 250 ppm. All experiments were measured at 27°C. The chemical shift of the rINEPT methyl peak at 13.8 ppm was used as an internal reference. All samples were equilibrated for 30 min prior to measurements. The temperature was calibrated using methanol (37).

### Small- and wide-angle X-ray scattering

All SAXS and wide-angle X-ray scattering measurements were performed on a SAXSLAB Ganesha 300XL with a High Brilliance Microfocus Sealed Tube as the X-ray source. Beam shaping was initially done by the shaped multilayer and further collimated by three sets of four-bladed slits. The scattering was detected by a Pilatus detector. The samples were mounted in solid-sample holders (sandwiches) with precut mica windows and heated with a Julabo water bath to either 27°C (Table 1, models B–F) or 23°C (Table 1, model A). The measurements had a *q*-range of 0.012 to 0.67 Å^−1^ (SAXS) and 0.05 to 2.5 Å^−1^ (wide-angle X-ray scattering). Data reduction was done with the autoprocessing tool of SAXSGUI, and the 1D scattering spectra were generated by radial integration of the 2D scattering pattern over all angles. Peak assignments were validated by plotting *q* for each reflection against the order of the reflection and fitting it to a linear-regression function for the lamellar phases (supplemental Fig. S2). The hexagonal phases were assigned by fitting the reflections to the ratios of 1, √3, 2, √7, and 3 (38). Lamellar repeat distances (*d*) were calculated from the slope of the linear regression, and the error bars for the spacings shown in Figs. 3 and 5 were estimated from the standard deviation of the average of the *d* calculated from each of the reflections (*d* = *n*2π/*q_n_*, where *n* is the order of the reflection). Peak maxima were determined with the find-peak function in MATLAB.

## RESULTS AND DISCUSSION

We evaluated the effects of Lti30 on lipid self-assembly at varying hydration conditions, including the effects on lipid molecular dynamics, lipid self-assembly structure, and phase transitions. To investigate each of these aspects, we used a range of different model lipid systems for which the composition was adjusted to give different phase behavior (Table 1, Fig. 1C). We first identified conditions in which Lti30 forms electroneutral complexes with anionic lipid lyotropic phases. The hydration conditions were then varied either by changing the total water content or by changing the RH above the sample. In the latter case, we can directly relate the RH to the osmotic pressure (Π*_osm_*) of water:

Πosm=−1VwΔμw=RTVwln(RH100)

where *V_w_* (m^3^ mol**^−^**^1^) is the molar volume of water and Δμ*_w_* is the chemical potential of water (39).

### Dehydrin-lipid association: effects on lipid molecular dynamics

When the Lti30 protein was added to dispersions of negatively charged unilamellar vesicles, there is spontaneous aggregation and precipitation of lipid-protein complexes. From scattering experiments (supplemental Figs. S3–S7) on these coaggregates several different self-assembly structures similar to the pure lipid systems were observed, including the liquid crystalline lamellar phase (L_α_) (lipid systems A–C) and reversed hexagonal phase (H_II_) (lipid system D). Lipid-protein complex formation was only detected in lipid systems that contained anionic lipids, and no protein-lipid association was observed for purely zwitterionic PC lipid systems (lipid system E; supplemental Fig. S8). These findings of lipid-protein association are fully consistent with previous reports on dehydrin-induced vesicle aggregation in systems composed of Lti30 dehydrins and anionic phospholipid vesicles (24, 25). They are also in line with previous studies showing that aggregation is suppressed when the electrostatic attraction between Lti30 and anionic lipid membranes is screened by the addition of salt (25). From the ^1^H solid-state NMR spectra it was further concluded that there are no detectable differences in the intensity from the different species between the pure lipid systems and the lipid-protein coaggregates (supplemental Fig. S9), which would be the case if there were strong selectivity in the lipid uptake into the lipid-protein coassemblies. When sodium chloride was added to the lipid vesicle solution in the absence of protein at concentrations up to 90 mM, no aggregation of vesicles was observed. The observed vesicle aggregation and precipitation can therefore not simply be explained by the screening of electrostatic interactions caused by the Lti30 polyelectrolyte and its counterions.

The association of Lti30 to anionic lipid membranes relies on a strong electrostatic attraction between the negatively charged lipid headgroups and the histidine-flanked K-segments in the Lti30 protein. In aqueous solutions, the Lti30 dehydrin protein is unstructured. It has previously been shown that when this protein associates with a negatively charged membrane, it goes through a conformational change in which the histidine-flanked K-segments of the protein locally fold up into α-helical segments and locate in the membrane headgroup interfacial layer (24–26) (Fig. 1A, B; supplemental Fig. S10). The uncharged segments in between the K-segments are likely unstructured and extend out from the membrane and into the solution. From a membrane perspective, the protein association will likely affect the packing and the molecular mobility of the lipid molecules, and it may also influence the self-assembly structure. Depending on the location of the protein in the bilayer and the protein-lipid interactions, different parts of the lipid molecules will be affected by the protein association. The consequences of the Lti30-lipid association on the molecular properties of the membrane lipids were here investigated using polarization transfer solid-state NMR (PT ssNMR) (40, 41). The NMR experiments provide information on molecular mobility in different carbons of the lipid molecules with close to atomic resolution, making it possible to distinguish what parts of the lipids are affected by protein adsorption. The PT ssNMR experiments are based on natural-abundance ^13^C solid-state NMR and involve the use of two common techniques to enhance the ^13^C signal: rINEPT and CP (34–36). The PT ssNMR method takes advantage of the fact that the transfer of polarization from the more abundant ^1^H nuclei to the ^13^C nuclei is sensitive to the molecular dynamics. The relaxation rates are determined by the reorientational motion and order of the C-H bond. For the more solid and/or ordered parts of the molecule, relaxation rates increase and rINEPT becomes inefficient. For such rigid parts, CP is efficient and thus gives a higher signal than rINEPT. The DP experiment does not involve polarization transfer, and the DP signal intensity may thus be used as a reference compared with the rINEPT and CP signal intensities. The rINEPT and CP signal intensities depend both on the rate and anisotropy (order parameter S_CH_) of the C-H bond reorientation (41), and the intensities can therefore not be directly used as quantitative measures of the molecular dynamics. The analysis of the data for changes in lipid molecular dynamics is therefore based on comparisons between the signal intensities from the rINEPT and CP pulse sequences relative to the signal obtained in the DP experiment rather than on the absolute intensities in each experiment.

**Fig. 2.**
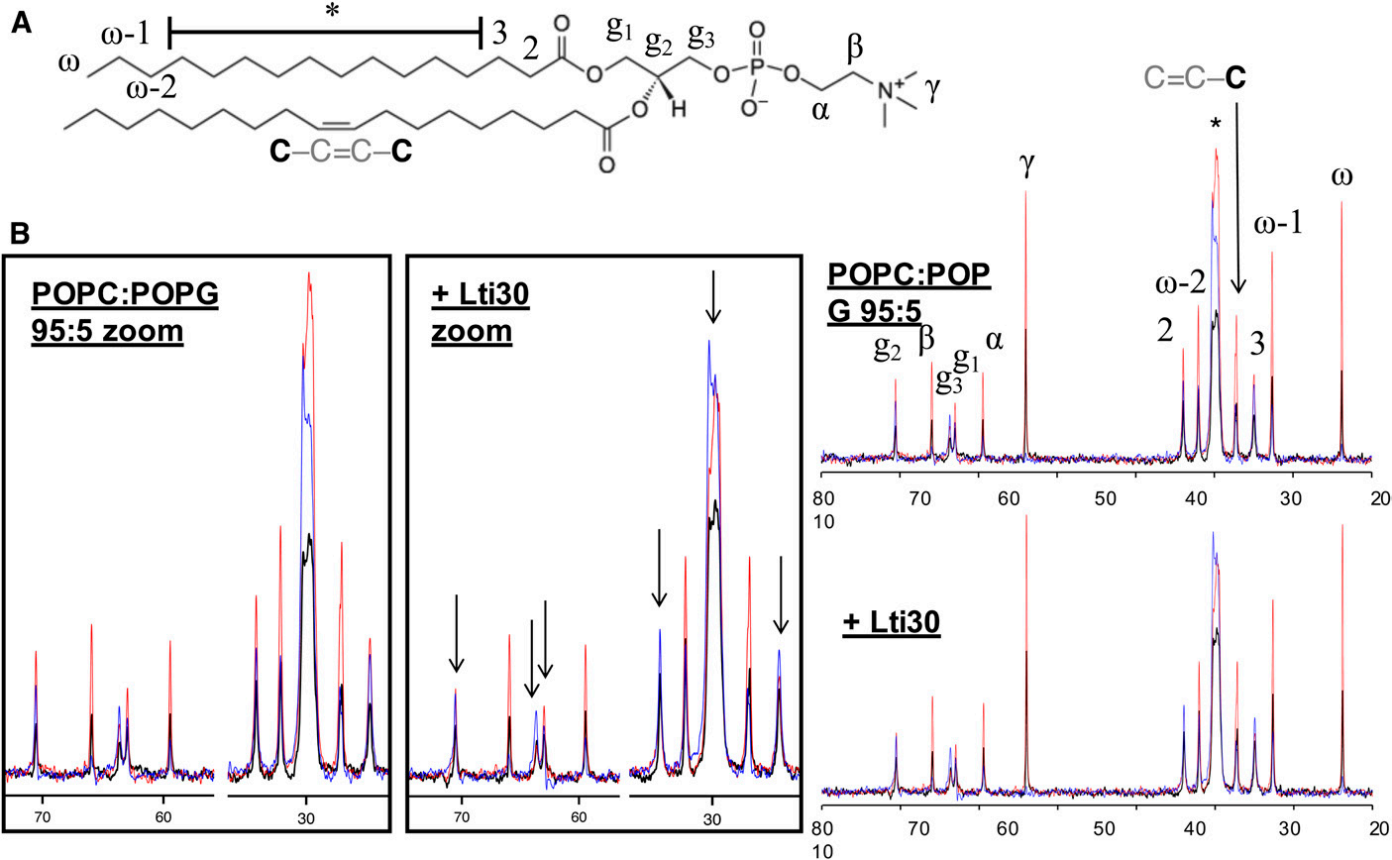
A: Chemical structure of POPC with numbered segments. B: ^13^C DP-CP-rINEPT spectra of the POPC:POPG (95:5) lamellar phase in the presence and absence of Lti30, with zoom-in pictures on the spectral regimes 22–35 ppm and 58–72 ppm. Arrows indicate peaks corresponding to the lipid carbons that are clearly affected by the addition of Lti30. Each graph shows three spectra: CP, blue; DP, black; and rINEPT, red.

**Figure 2** shows PT ssNMR spectra measured from the lipid model system POPC:1-palmitoyl-2-oleoyl-*sn*-glycero-3-phosphoglycerol (POPG) (Table 1, lipid system A) in the presence and absence of Lti30. Both samples form a lamellar bilayer phase as confirmed by SAXS (supplemental Fig. S3). Both spectra are characteristic of the liquid crystalline lamellar phase, L_α_, giving rise to both rINEPT and CP signals for all carbon atoms in the lipid molecule (40, 41). A closer inspection of the spectra revealed that the addition of Lti30 led to a clear reduction of the rINEPT intensity from the carbons in the glycerol backbone (g2 and g3; Fig. 2B) as well as carbons in the acyl chain. The single most affected carbon is the g3, which is next to the negatively charged phosphate group. The carbons in the acyl chain that are most strongly affected are those that are located closest to the interface (C2, C3, and the crowded spectral region marked with an asterisk in Fig. 2B; supplemental Fig. S11). The crowded spectral region contains a signal from carbons in the acyl chain from C4 to C13 that cannot be resolved from these experimental data, and therefore we cannot draw any conclusions on which of these carbons are most affected by the addition of the protein. It is further concluded that there are less detectable changes in the intensity of the rINEPT signal relative to the CP and DP signals for the rest of the lipid acyl chain, implying that the protein does not affect the parts of the lipids that are buried in the center of the bilayer nearly as much. Due to the low abundance of PG lipids, it was not possible to resolve the effects on the separate lipid components. We were only able to resolve the peak from the carbonyl carbon in the protein backbone in the DP NMR spectra and we could not resolve any other carbons from amino acids, which can be explained by the low concentration of each amino acid in the lipid-protein complex. In summary, from the NMR data in Fig. 2, we draw the conclusion that the membrane-bound protein mainly affects the lipids in the region close to the bilayer interface, which is fully consistent with the previously proposed structure of the protein located in the interfacial layer strongly interacting with the lipid headgroups (25). The observed reduced mobility in parts of the lipid acyl chain close to the headgroup can be explained by strong interactions in the interfacial layer, which will also influence dynamics in the neighboring parts of the lipid molecules. There are no clear effects on the lipid acyl chains deeper inside the bilayer, which would be expected if the protein were penetrating deeper into the membrane. Here we notice structural similarities to the systems composed of α-helical DNA compacted in an oppositely charged lipid or surfactant self-assembled systems (42–44), which leads to a reduction in lipid molecular dynamics (40, 45).

### Dehydrin makes the lipid lamellar system less sensitive to changes in water content compared with the protein-free lipid system

**Fig. 3.**
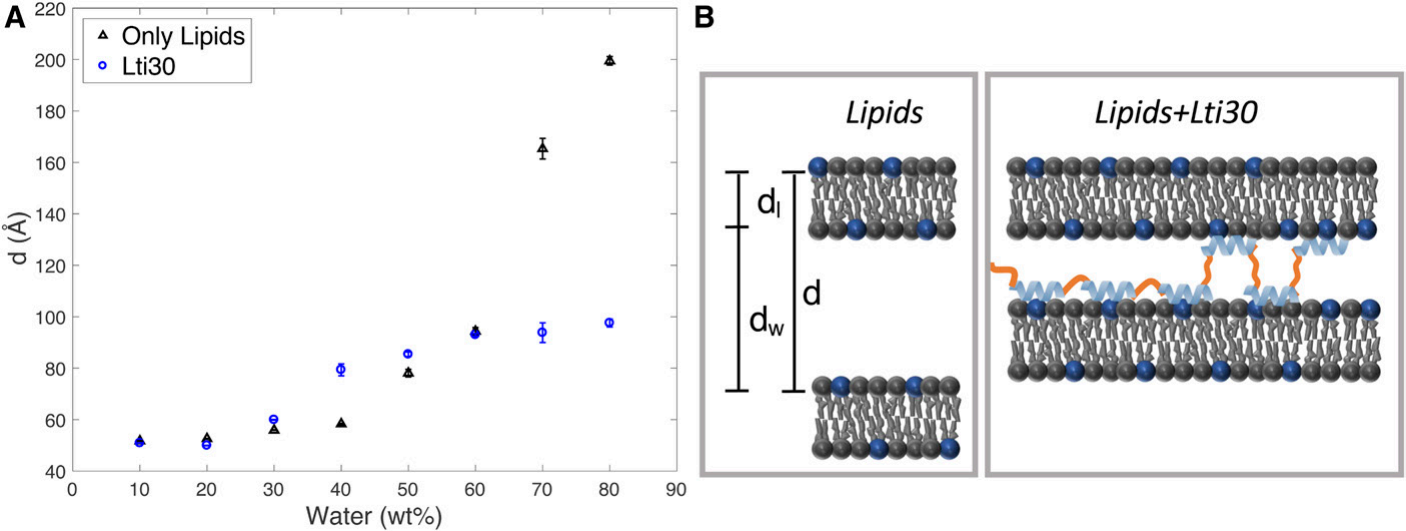
A: Lamellar repeat distance, *d*, measured for the POPC:POPG (95:5) lamellar phase at varying water content for the system composed of lipids alone (black) and lipid-protein complexes containing Lti30 (blue). B: Schematic illustrations of the suggested molecular arrangement in the hydrated pure lipid system and hydrated lipid-protein complex, respectively.

Next, we investigated whether the dehydrin Lti30 protein may act to stabilize the lamellar membrane structure, making it less sensitive to variations in hydration conditions. For these studies, we chose the same model system as described above (POPC:POPG, lipid system A), which forms a liquid crystalline lamellar L_α_ phase over a large range of water contents. **Figure 3A** demonstrates a very strong effect of Lti30 on the lamellar swelling. In the lipid systems with no added protein (black symbols), there was a dramatic increase in lamellar repeat distance with increasing water content, which was due to the electrostatic repulsion between the charged lipid headgroups. For the current system, the interbilayer separation, *d_w_*, increases from ∼46 Å to ∼170 Å when going from 50 to 80 wt% water, assuming that the thickness of the hydrocarbon layer of the bilayer, *d_l_*, does not change over the whole range of water contents [*d_l_*= 27.1 Å (46)]. In the limit approaching full hydration, *d_w_* is up to six times thicker compared with the hydrophobic part of the bilayer, *d_l_*. The swelling profile is completely different for the lamellar phase that contains the Lti30 protein (Fig. 3A, blue symbols; supplemental Figs. S3–S6), which shows a rather shallow swelling profile. The data clearly show that the addition of protein influences the lamellar swelling both at high and low water contents. The maximum swelling of the dehydrin-containing lamellar phase is reached at ∼60 wt% water, with a lamellar repeat distance of 94 Å and an aqueous layer separation *d_w_* of 67 Å. At higher water contents, the fully swollen lamellar phase coexists with an excess aqueous solution. At water contents below 60 wt%, the measured thickness of the interbilayer aqueous separation is slightly larger for the dehydrin-containing lamellar phase compared with the protein-free lipid lamellar system at the same water content. Overall, the addition of Lti30 gives rise to a more robust lamellar system that is less sensitive to variations in water content than the protein-free system, which may have important consequences on preventing membrane reorganization induced by the hydration or dehydration of cellular systems.

The dehydrin Lti30 protein consists of the membrane-bound positively charged K-segments, which are separated by uncharged unstructured segments (25). One can envision that the protein adsorbs to the negatively charged membrane interface with the K-segments bound and the uncharged segments extending into the solution (Fig. 1A, B; supplemental Fig. S10). In the multilamellar system with the Lti30 protein with multiple binding K-segments, the unstructured segments may either fold back to the same bilayer or bridge the adjacent bilayers (Fig. 3C). We can estimate on the basis of the structure of Lti30 (Fig. 1A, B) that the length of the uncharged segments that contain 8–19 amino acids is 26–62 Å, assuming a coil structure. If the unstructured segment were fully extended, the total length would be 30–72 Å. The maximum interbilayer separation, *d_w_*, obtained from the SAXS data shown in Fig. 3A is thus similar to the length of the longer unbound segments. For the shorter segments, the probability of folding back to the same bilayer is higher compared with the longer segments, which may explain why the interlamellar separation is determined by the length of the longer segments. It is noted that the calculated value of *d_w_* contains both the region of the polar headgroups and interbilayer aqueous layer, and it is therefore reasonable that this value is slightly larger than the length of the unstructured segments of the protein. The bridging behavior of analogues has previously been described for polymer-surfactant systems with flexible hydrophilic chains flanked with segments that associate with the bilayer either through electrostatic or hydrophobic interactions (47–50).

### Dehydrin influences dehydration-induced lipid phase transitions

**Fig. 4.**
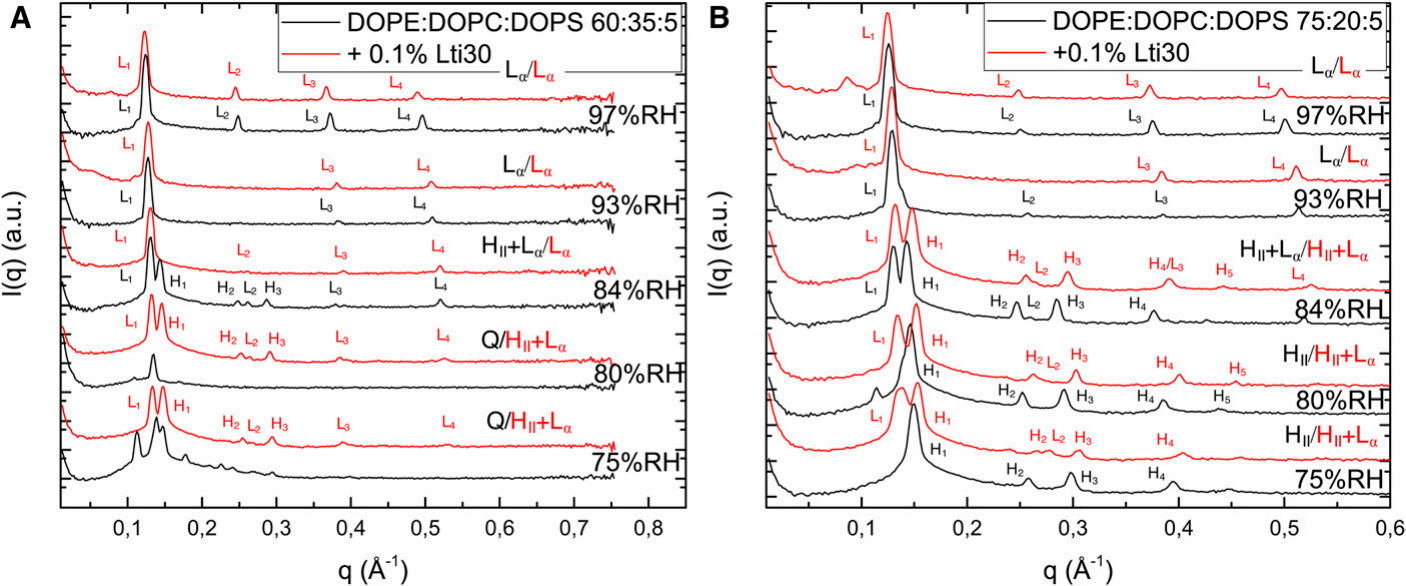
A: SAXS spectra of DOPE:DOPC:DOPS (60:35:5l; black) and with added Lti30 (red) at varying RHs. B: SAXS spectra of DOPE:DOPC:DOPS (75:20:5; black) and with added Lti30 (red) at RHs between 75% and 97%.

When the lipid system is exposed to an increased osmotic pressure, there will naturally be reduction in the water content and deswelling of the lamellar phases. In many lipid systems, dehydration will also lead to phase transitions, which may in turn have consequences on membrane integrity and functions (51). Here, we tested the hypothesis that dehydrin can stabilize the fluid bilayer L_α_ structures, which might be a way to protect the membrane system against drying-induced injury. We studied the effect of dehydrin on phase transitions between lamellar and nonlamellar structures using model lipid systems with a composition that is relevant to the compositions of some cellular plasma membranes in seeds and leaves (1, 52, 53) (Table 1, lipid systems B and C) and characterized phase behavior in the presence and absence of protein by means of SAXS. The charged PS lipids were added to promote electrostatic attraction and complex formation. For the ternary dioleoylphosphatidylethanolamine (DOPE):dioleoylphosphatidylcholine (DOPC):dioleoylphosphatidylserine (DOPS) lipid mixture, dehydration resulted in structural transformations from planar bilayers to closely packed rods (H_II_ phase) or folded bilayers in a bicontinuous cubic phase (Q) depending on the proportions of the different lipid components (**Fig. 4**, black curves). From the current scattering patterns, we could not assign the space group for the bicontinuous cubic phase. For both lipid systems investigated, the addition of dehydrin Lti30 (Fig. 4, red curves) had a strong effect on the self-assembly structure, and the planar bilayer L_α_ structure was stable in dry conditions, in which the nonlamellar H_II_ or Q phases were present in the absence of dehydrin Lti30. In other words, the addition of dehydrin Lti30 can stabilize the planar bilayer phase in favor of nonlamellar phases. This may be crucial in maintaining the membrane integrity to prevent massive leakage and cell injury (1, 2).

### Dehydration-induced phase transitions: comparison between dehydrin Lti30 and small osmolytes

Nature has developed different molecular strategies to protect its biomolecules against desiccation. Dehydrin proteins can be seen a sophisticated class of protection molecules in that this macromolecule explores different conformational states in its free and membrane-bound states (24–26). Far simpler approaches found in nature involve the use of small polar (osmolyte) molecules, such as sugars, free amino acids, urea, glycerol, and TMAO (13, 14, 16, 18, 50, 54–57). The mechanism by which these small molecules protect the biological systems against osmotic stress is different from that of the dehydrins and may also be different between the different osmolyte compounds (29, 32, 58). The last question addressed in this article concerns the similarities and differences between dehydrin Lti30 and small polar osmolytes in their effects on lipid membrane systems at varying hydration conditions. For these studies, we chose two model osmolytes, urea and TMAO, which were added to the model lipid mixtures for which dehydration leads to a transition between lamellar and nonlamellar phases (Table 1, lipid system B). These model osmolytes are not common in plants, and they were chosen because they are already well characterized in similar lipid systems (29–32).

**Fig. 5.**
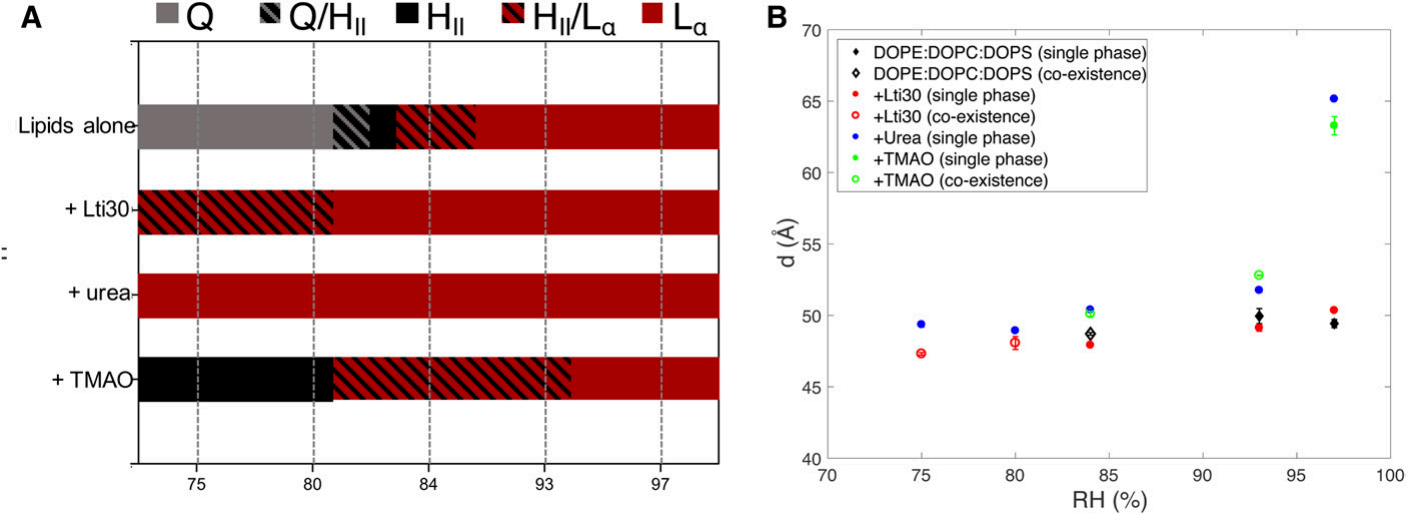
A: Summary of how Lti30, urea, and TMAO influence lipid phase behavior of the DOPE:DOPC:DOPS (60:35:5) system at varying RHs. The neat lipid system forms an L_α_ phase (red) at high RHs. At lower RHs, the same lipids form an H_II_ phase (black) and bicontinuous cubic phase (gray). The regions with stripes indicate phase coexistence. The addition of 0.1 mol% Lti30 to the lipid system makes the L_α_ phase stable at lower RHs compared with the neat lipid system, with a single L_α_ phase present at RHs down to 80% and coexisting L_α_/H_II_ phases at lower RHs. The addition of 5 wt% urea to the same lipid makes the L_α_ phase stable over the entire range of RHs investigated. When 5 wt% TMAO is added to the same lipid system, the phase boundaries are moved to higher RHs, and the H_II_ phase is stable at higher RHs compared with the neat lipid system. The dashed lines represent measured data points for which the phase behavior was characterized, and the one- and two-phase regions indicated in between the measurement points were deduced from thermodynamic arguments to fulfill the phase rule. The exact positions of the phase boundaries were not determined. B: Lamellar repeat distance of the L_α_ phase in the DOPE:DOPC:DOPS (60:35:5) system (black) with either added Lti30 (red), urea (blue), or TMAO (green). Filled symbols indicate a single L_α_ phase; open symbols indicate that the L_α_ phase coexists with an H_II_ phase.

The results from the comparative studies for dehydrin Lti30, urea, and TMAO are summarized in **Fig. 5**. We first compared Lti30 with urea in dry conditions. Although these molecules and their molecular actions are widely different, they both can prevent the transition to nonlamellar phases at dry conditions, and the lamellar phase is present at RH values at which the neat lipid system would form a cubic or hexagonal phase (Fig. 5A). We have previously investigated the structural and thermodynamic consequences of adding osmolytes such as urea and glycerol to lamellar phospholipid membranes in dry conditions (29–31). When added to the lipid system, these small polar molecules reduce the osmotic pressure while behaving in a neutral way with respect to lipid membrane self-assembly (29–31, 59). In dry conditions, the water content will decrease, while the small polar compounds with low vapor pressure will remain in the system and contribute to the volume of the polar regions. In this way, the osmolytes can in an unspecific way substitute for the water in such a way that properties of the lipid lamellar system remain largely unchanged (29–31). This can be compared with the “water replacement hypothesis” for sugars in lipid membranes presented by Crowe et al. (56), which assumes a direct headgroup-solute interaction, whereas in the cases of, for example, urea and glycerol, we have previously proposed an unspecific mechanism in which the properties of lipids appear neutral relative to the replacement of water with the solute in the liquid phase (29–31). By exchanging urea for TMAO, we then illustrate the importance of specific interactions between the solute and the lipid headgroups, which becomes important at low water contents. Through combined experimental and molecular dynamic simulation studies, it has been shown that TMAO is depleted from the aqueous solution close to the phosphatidyl choline headgroups (31, 32). This depletion will lead to a less hydrated headgroup layer in the presence of TMAO (32), which may in turn explain why the H_II_ structure with a negative curvature is favored above the planar L_α_ structure.

Finally, we compared the effects of Lti30 and the simple small osmolytes at high relative humidities (Fig. 5B). Here, we have shown that Lti30 can act to prevent massive swelling of the lamellar system (Fig. 3), likely due to interbilayer bridging. This is in contrast to the small molecules urea or TMAO, which both cause extensive swelling of the lamellar phases in the same conditions. These and other polar solutes generally affect only the self-assembly through the aqueous solution, and they will not prevent extensive swelling in humid conditions unless they are completely depleted from the liquid crystalline phase (32).

### Biological and technical implications

A major challenge to plant growth, productivity, and survival are the changes in temperature and vanishing supply of water. How plants adjust to such osmotic stresses is gaining increased interest in the expansion of territories suitable for food production. In acute stress, plants express stress proteins, such as dehydrins, which seem to protect and maintain the integrity of the cell membranes. In this article, we investigated how dehydrin proteins may protect membranes against osmotic stress. The results presented here corroborate the idea of a sensitive and multifaceted regulatory mechanism of dehydrin function in stressed plant cells. The lipid interaction of Lti30 provides not only a base for mechanistically elucidating the stress defense of plant cells but also points to the possibility that the functional repertoire of the dehydrin proteins is larger than previously anticipated.

In nature, several protection systems often work in parallel, including both dehydrins and small polar molecules. Here, we showed that both dehydrins and osmolytes may prevent dehydration-induced structural changes in dehydrated conditions and that dehydrins may also prevent extensive swelling at high water contents. Compared with many other proteins, LEA proteins and dehydrins are also soluble and stable at a high osmotic pressure, where they tend to increase their helical content (60–65). These proteins may therefore also be functional in extreme and dry conditions. In addition to the fundamental understanding of how LTi30 influences membrane structure and dynamics, insights into different strategies to protect dry lipid and protein systems can be valuable for various technical applications, such as the stabilization of dry lipid-based formulations in pharmaceutical and food applications.

## CONCLUSIONS

Most organisms in nature are in one way or another affected by osmotic stress. At the cellular level, this can lead to structural reorganization in macromolecular assemblies that can have severe consequences on biological functions. Many plants and seeds are exposed to dry and cold climates or soil with high salinity. In acute stress, plants express stress proteins, such as dehydrins, that are thought to protect and maintain the integrity of the cell membranes. In this article, we investigated how the dehydrin Lti30 protein may act to stabilize membrane systems that are exposed to large variations in hydration conditions. We also compared the effects of dehydrins to those of some simple osmolyte molecules. The overall picture that emerges from these studies is that there is a strong association between dehydrin Lti30 and anionic lipid membranes. The mixed lipid-protein system forms self-assembled structures similar to those found in the protein-free system, although the self-assemblies are less sensitive to changes in hydration conditions. This is manifested through the depression of dehydration-induced phase transitions and the reduced swelling of multilayer bilayer systems. In other words, the lipid-protein system appears more robust and stable to changes in the outside-environment hydration conditions, which might be crucial to the protective role of dehydrin Lti30 in drying membrane systems. The main conclusions from the experiments presented here are as follows:

The association of Lti30 with anionic lipid membranes relies on a strong electrostatic attraction between the negatively charged lipid headgroups and the positively charged α-helical parts of the dehydrin protein. We conclude on the basis of the PT ssNMR experiments that the membrane-bound protein mainly affects the lipids in the region close to the bilayer interface. This finding is consistent with the finding that the protein located within the interfacial layer strongly interacts with the lipid headgroups (25). From the current data there are no indications of major perturbations of the lipids deeper into the bilayer.

The dehydrin Lti30 protein acts to stabilize lamellar multilayer structures, making them less sensitive to variations in hydration conditions compared with the protein-free system. This may be a protective mechanism that prevents membrane rupture induced by osmotic swelling in cellular systems. The proposed molecular explanation relies on the protein bridging adjacent bilayers in a multilayer bilayer system in which the bridging part consists of unstructured protein segments in between the membrane-bound α-helical parts (Fig. 3C).

In the model lipid system with a composition relevant to many seeds and plants, dehydrin has a remarkable effect of making the lamellar bilayer phase robust toward changes in hydration conditions, preventing the formation of nonlamellar phases upon drying. This may be crucial in maintaining the membrane integrity to prevent massive leakage and cell injury.

The effects of dehydrin Lti30 on lipid self-assembly in varying hydration conditions were compared with the effects of small polar model osmolytes, urea, and TMAO. We showed that Lti30 dehydrin can suppress phase transitions at dry conditions and prevent extensive swelling at high water contents. The small polar urea, which is not depleted from the bilayer interface, can also prevent dehydration-induced phase transitions but cannot prevent massive swelling of the hydrated structures in conditions excess swelling. The polar solutes affect only the self-assembly though the aqueous solution, and they will not prevent extensive swelling in humid conditions.

### Data availablity

All the data described in the article are contained within the article.

## Supplementary Material

Supplemental Data
