# Supplementary material for: The plant dehydrin Lti30 stabilizes lipid lamellar structures in varying hydration conditions
Source: J Lipid Res. 2020 May 13;61(7):1014–24. doi: 10.1194/jlr.RA120000624 (PMC7328047; doi:10.1194/jlr.RA120000624)
Supplement: Supplemental Data [file supp_61_7_1014__index.html]

The plant dehydrin Lti30 stabilizes lipid lamellar structures in varying hydration conditions — Dehydrin Lti30 stabilizes lipid lamellar structures — The plant dehydrin Lti30 stabilizes lipid lamellar structures in varying hydration conditions — Supplemental Data 

# The plant dehydrin Lti30 stabilizes lipid lamellar structures in varying hydration conditions

## Supplemental Data

- SI - Supplementary figures
