# Supplementary material for: The plant dehydrin Lti30 stabilizes lipid lamellar structures in varying hydration conditions
Source: J Lipid Res. 2020 May 13;61(7):1014–24. doi: 10.1194/jlr.RA120000624 (PMC7328047; doi:10.1194/jlr.RA120000624)
Supplement: Supplemental Data [file supp_RA120000624_158168_2_supp_528262_qm6m66.pdf]

THE PLANT DEHYDRIN LTI30 STABILIZES LIPID LAMELLAR STRUCTURES IN VARYING HYDRATION CONDITIONS

Jenny Marie Andersson[1], Quoc Dat Pham[1], Helena Mateos,[1] Sylvia Eriksson[2], Pia Harryson[2], Emma Sparr[1*]

[1] Division of Physical Chemistry, Chemistry Department, Lund University, P.O. Box 124, 22100 Lund, Sweden

[2] Department of Biochemistry and Biophysics, Stockholm University, Svante Arrhenius väg 16C, 106 91 Stockholm, Sweden

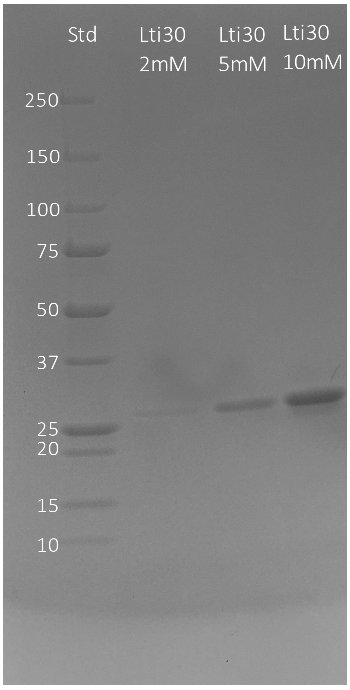

Supplementary Figure S1. SDS gel of purified Lti30 after freeze dried and dissolved in 5mM Mes buffer at three different concentrations 2mM, 5mM and 10mM. First lane is protein standard (Std) (Biorad, Precision plus protein standards, from bottom on gel: 10,15,20,25,37,50,75,100,150,250KDa) .

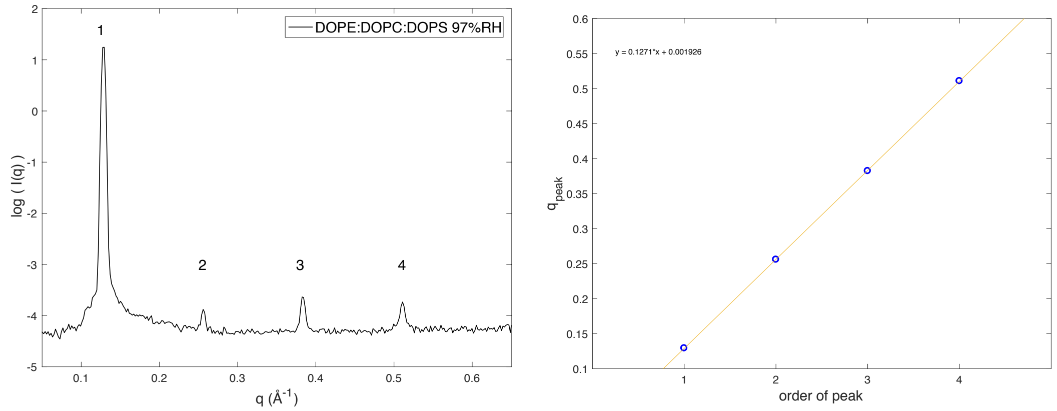

Supplementary Figure S2. The assignment of peaks is validated by plotting q of each peak against the order of the reflection of each peak and checking that they can be fitted with a linear regression function. The lamellar repeat distance (d) is extracted from the slope of the curve and error bars are estimated from the standard deviation on the average of d calculated from each reflection.

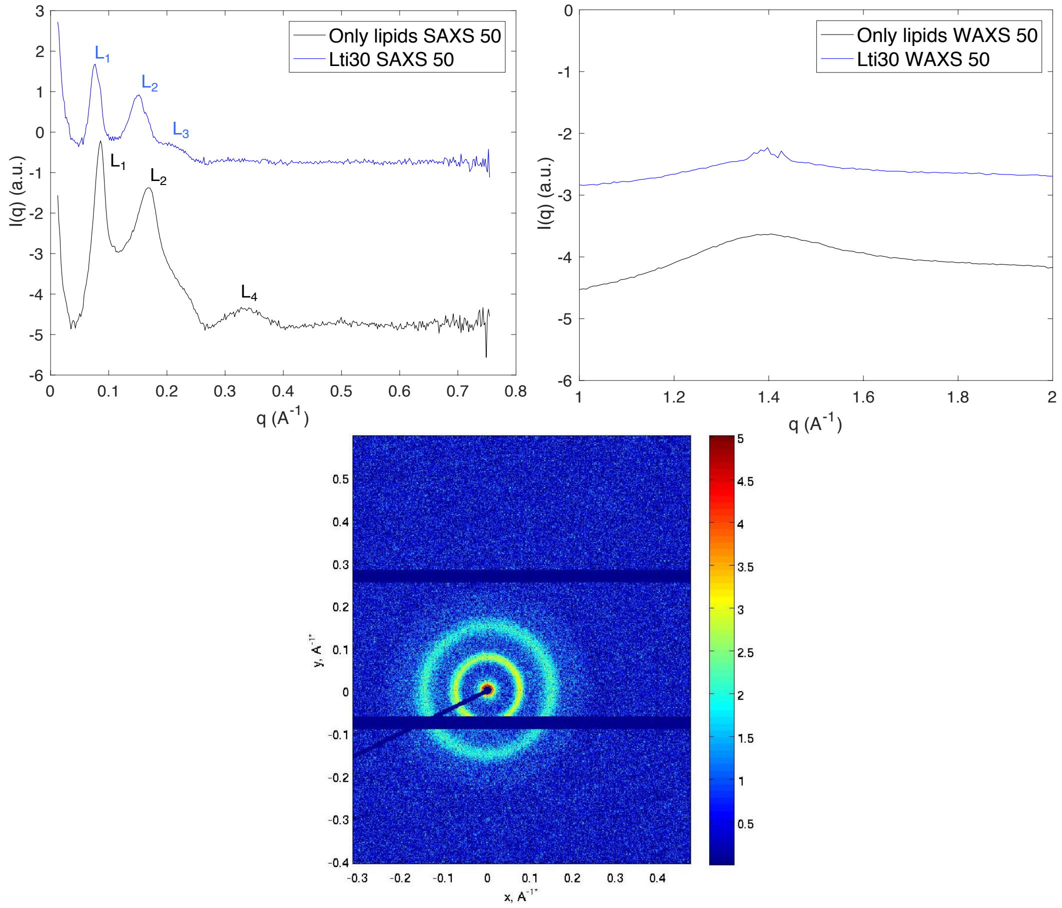

Supplementary Figure S3. SAXS and WAXS spectra of POPC:POPG 95:5 (black) and POPC:POPG 95:5 + 0.1% Lti30 (blue) at 50 wt% water, together with an example of isotropic 2D spectrum for the sample containing DOPC:DOPG+Lti30 and 50 wt% water.

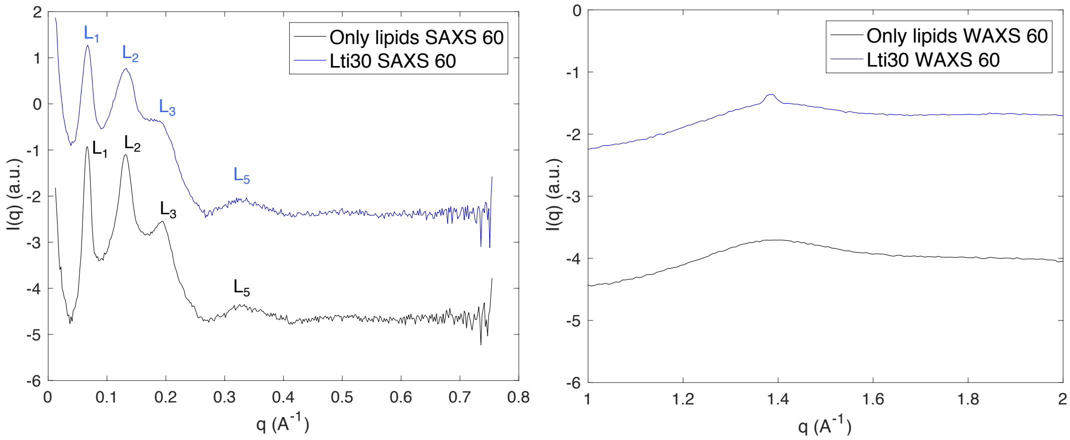

Supplementary Figure S4. SAXS and WAXS spectra of POPC:POPG 95:5 (black) and POPC:POPG 95:5 + 0.1% Lti30 (blue) at 60 wt% water.

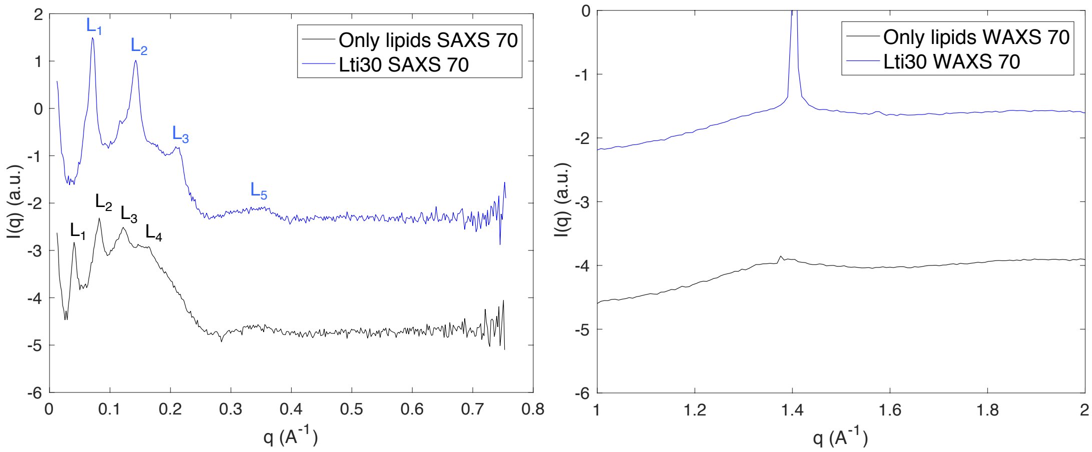

Supplementary Figure S5. SAXS and WAXS spectra of POPC:POPG 95:5 (black) and POPC:POPG 95:5 + 0.1% Lti30 (blue) at 70 wt% water. The sharp peaks in the WAXS spectra originate from the mica window and should not be considered in the evaluation.

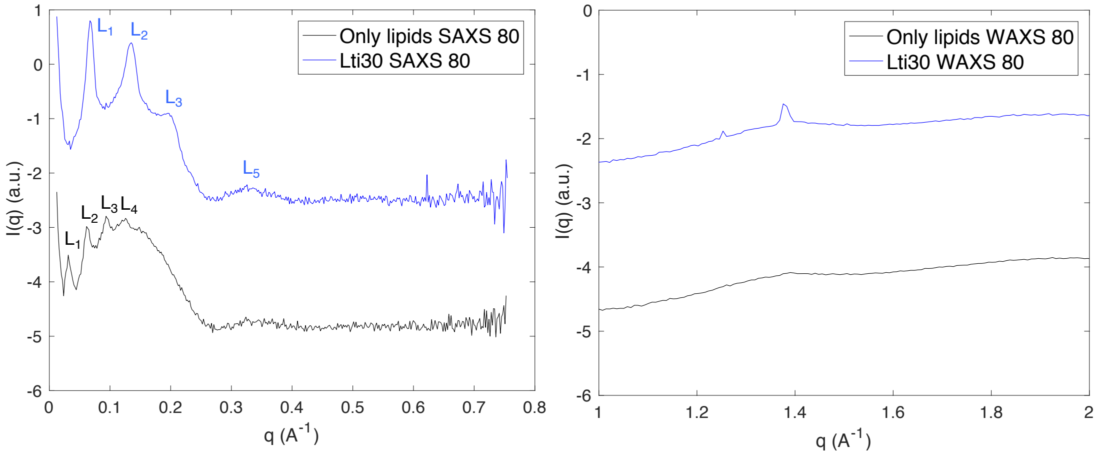

Supplementary Figure S6. SAXS and WAXS spectra of POPC:POPG 95:5 (black) and POPC:POPG 95:5 + 0.1% Lti30 (blue) at 80 wt% water. The sharp peaks in the WAXS spectra originate from the mica window and should not be considered in the evaluation.

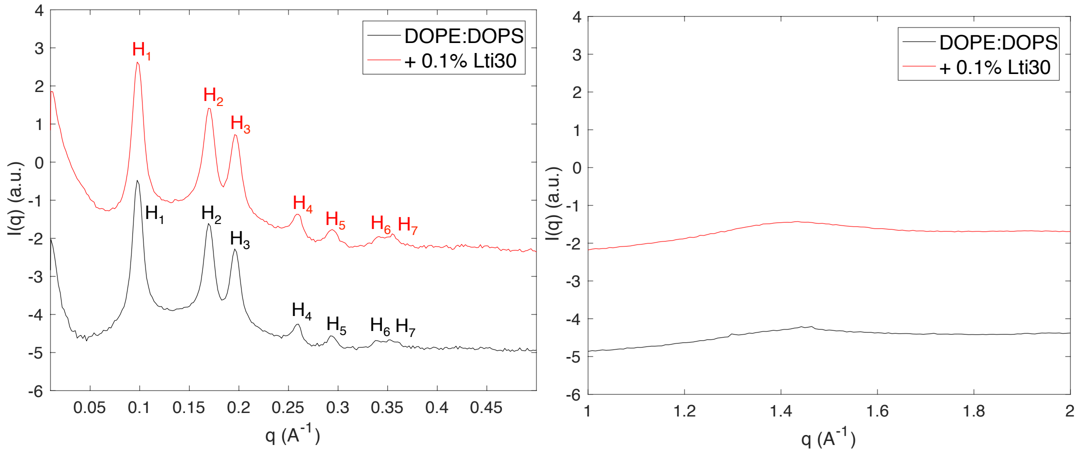

Supplementary Figure S7. SAXS and WAXS spectra of DOPE:DOPS 95:5 (black) and + 0.1% Lti30 (red) in excess water.

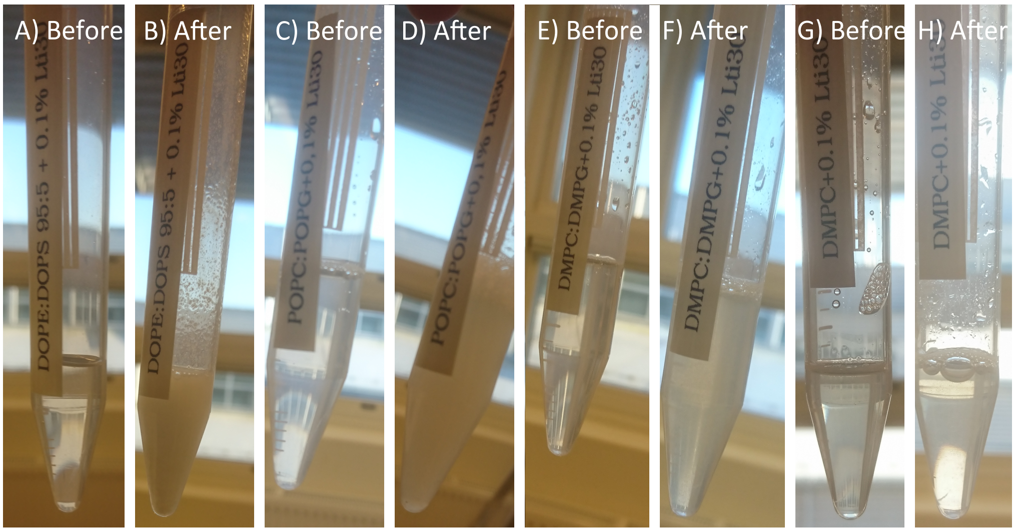

Figure S8. A) Vesicle solution of DOPE:DOPS 95:5 before addition of Lti30 and B) after addition of 0.1% Lti30. C) Vesicle solution of POPC:POPG 95:5 before addition of Lti30 and D) after addition of 0.1% Lti30. E) Vesicle solution of DMPC:DMPG 95:5 before addition of Lti30 and F) after addition of 0.1% Lti30. C) Vesicle solution of DMPC before addition of Lti30 and D) after addition of 0.1% Lti30.

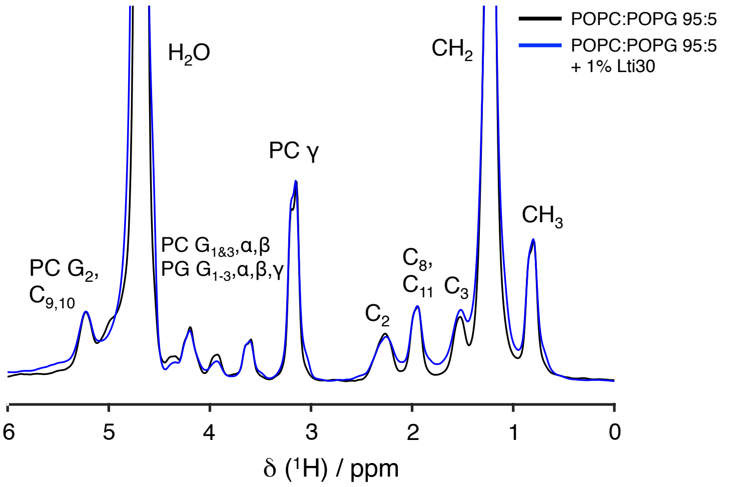

Supplementary Figure S9: ^{1}H ssNMR spectra of POPC:POPG 95:5 (black) and POPC:POPG 95:5 + 1% Lti30 protein (blue) normalized by the CH$_3$ peak at ~0,8 ppm which is the same for all lipids in the sample. The peaks from the majority of the carbons in the lipid head groups from both PC and PG overlap in the ppm region between 3,4 - 4,6 ppm. The intensity profile of all peaks in this region remains the same between the two samples with and without protein, showing that there are no significant changes in lipid composition remained upon complexation with dehydrin.

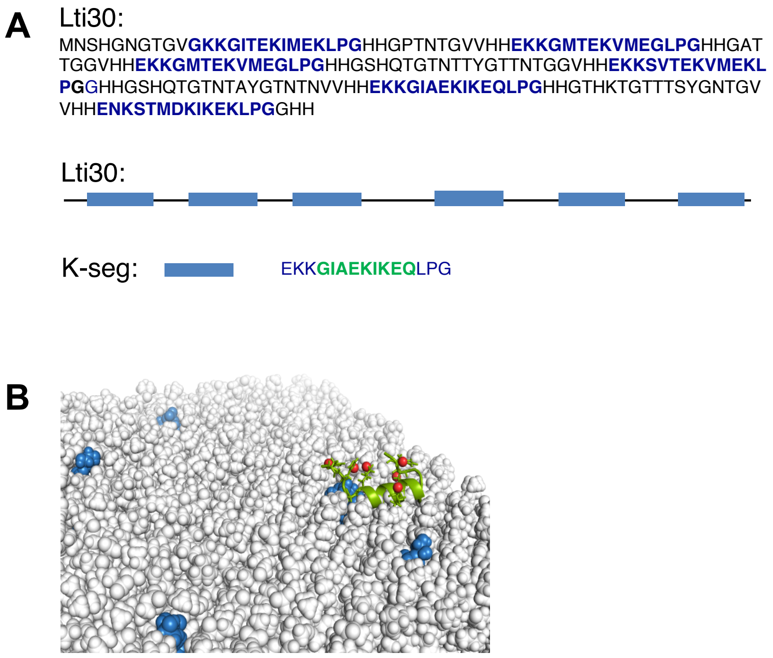

Supplementary Figure S10. A) Amino acid sequence of Lti30 with K-segments written in dark blue. Schematic picture on K- segments (blue box) distribution along the sequence. K-segment with the helix forming amino acids in green. B) Model of K-segment binding to a lipid surface (PC:PG). [1]

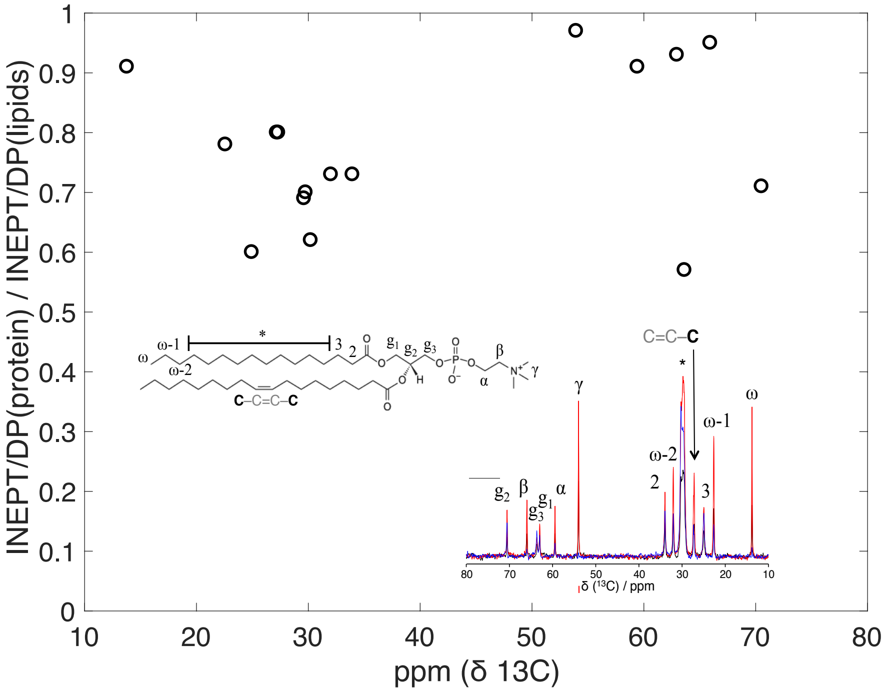

Supplementary Figure S11. Relative changes in the INEPT/CP intensity ratios in samples contain both lipids and protein compared to samples composed of only lipids. The insert spectra are used for the peak assignment.

Reference

1. Eriksson, S.; Eremina, N.; Barth, A.; Danielsson, J.; Harryson, P., Membrane-Induced Folding of the Plant Stress Dehydrin Lti30. *Plant Physiol* **2016,** *171*, 932–943.
